# Poisson Mixture Regression Models for Heart Disease Prediction

**DOI:** 10.1155/2016/4083089

**Published:** 2016-11-23

**Authors:** Chipo Mufudza, Hamza Erol

**Affiliations:** Statistics Department, Cukurova University, 01330 Adana, Turkey

## Abstract

Early heart disease control can be achieved by high disease prediction and diagnosis efficiency. This paper focuses on the use of model based clustering techniques to predict and diagnose heart disease via Poisson mixture regression models. Analysis and application of Poisson mixture regression models is here addressed under two different classes: standard and concomitant variable mixture regression models. Results show that a two-component concomitant variable Poisson mixture regression model predicts heart disease better than both the standard Poisson mixture regression model and the ordinary general linear Poisson regression model due to its low Bayesian Information Criteria value. Furthermore, a Zero Inflated Poisson Mixture Regression model turned out to be the best model for heart prediction over all models as it both clusters individuals into high or low risk category and predicts rate to heart disease componentwise given clusters available. It is deduced that heart disease prediction can be effectively done by identifying the major risks componentwise using Poisson mixture regression model.

## 1. Introduction

Heart disease encloses a number of conditions that influence the heart and not just heart attacks. These may include functional problems of the heart such as heart-valve abnormalities, high blood pressure (BP), smoking, diet, cholesterol, or irregular heart rhythms. Problems like these can lead to heart failure, arrhythmia, and a host of other problems. The work in [1] claims that heart diseases have become the leading global death accounting for 17.3 million deaths per year and killing 1 in 7 in the US alone. Therefore effective and efficient automated heart disease prediction systems can be beneficial to both the patient and cardiologist. Although there has been increasing interests on heart disease problems especially with the use of data mining techniques and algorithms, most of them concentrated on a supervised classification approach through different classifications and algorithms.

In a comparative approach research by Bagirov et al. [2] they showed that it is possible to classify heart disease problems using either supervised or unsupervised classifications. Supervised classification on different patients status by data mining algorithms to predict heart disease has been explored by various authors in machine learning. H. D. Masethe and M. A. Masethe [3] identified that the predictive accuracy of J48, over REPTREE and CART, was reliable for heart disease prediction in South Africa. Weighted fuzzy models based on supporting have also been studied and analysed showing an improvement against the network based models [4]. Supervised classification algorithms can improve the efficiency to cardiologist as shown by Taneja [5] on PGI data where over 7300 observations were classified using J48 and Naive Bayes in WEKA. In a general review on the heart disease using data mining techniques done by Kaur and Singh [6], they summarised that most researches show that the main risk factors are cholesterol, lack of exercise, obesity, and high blood pressure whilst the best algorithms seem to be dominated by decision trees.

Supervised classifications predictions can also be improved in some cases by incorporating unsupervised classification techniques like clustering as a preprocessing procedure. However, it may not always be the case as shown by Soni et al. [7] that decision trees can still outperform the Bayesian classification even when clustering is incorporated although they mentioned that both algorithms can be improved by genetic algorithms. They also implemented a combination of associative classification and genetic algorithms in an effort to come up with the best system of heart disease prediction. A similar result by Akhil et al. [8] shows out that J48, a decision tree based algorithm, outperforms the Naive Bayes and neutral network on a different data set for heart disease prediction. Different data mining techniques for diagnosis have achieved varying probabilities for unique methods. In general under supervised classification methods like decision trees, Naive Bayes, and neural networks, decision trees seem to be better classifiers for heart disease diagnosis [9–11]. Regression trees, ID3, and CART can also be used on heart diseases prediction based on the important variables which are chest pain, exercises induced (exang), slope, and resting sugar level (restecg) where CART tend to be best classifier [12].

Although supervised classification seems to be effective, real life data normally comes as a complex mixture of both continuous and categorical data variables together with a lot of missing information. This also comes with difficulties in data manipulations since well defined methods deal with either categorical or continuous data. Models involving mixed data generally experience variability and exhibit complex heterogeneous properties. Mixtures of categorical and continuous data are normally handled by generalised linear models (GLMs) of either Poisson or binomial maximum likelihood. However, in most cases these are normally not adequate enough to capture all the heterogeneity involved. GLMs in which the response is count (geometric, binomial/Poisson) are also sometimes referred to as count data problems. They appear in many real life settings, such as predicting the number items in economics, analysing infectious diseases in health and many modeling topics in the corporate world. Mixture models in a GLM context then become robust in capturing the heterogeneity where GLM cannot suffice. They allow the response for each observation *y*_*ij*_ to depend on *x*_*j*_ of the covariate vector where heterogeneity is exhibited and prior information is not available by grouping the likely homogeneous observations [13].

Mixture models are a form of unsupervised pattern recognition used to identify groups in the data based on a set of response variables or *y*-variable. Simultaneously they can be used to investigate relations of the groups themselves, or the within group expectations of the response variable given a set of covariates, or *x* variables. Count data mixture models often deal with extra dispersion problems especially in medical research where data is often count. As such there has been increasing interest in modeling count data using the Poisson process and geometric and negative binomial [14, 15]. We attempt to present a detailed study on heart disease prediction using mixture model based clustering techniques on count data. Poisson mixture regression models will be here applied to heart disease data in order to determine how different predictor variables affect the heart rate diagnosis in different groups, unknown but to be identified. Unsupervised classification, unlike in the previous works, will be here applied to determine heart disease prediction efficiently. Model based clustering will be here administered in a Poisson mixture regression model way. Although clustering has been incorporated in some classification work previously done, it was only incorporated as preprocessing step before classification. The predictions will be accomplished via Poisson mixture regression models in a GLM context and identification of the most important attributes (risk factors) for each cluster will be done through the mixture model, thus a way to effectively help reduce the number of tests needed by cardiologist to diagnose heart disease. Linear regression mixtures have also been studied extensively, especially when no information about membership of the points assigned to each line was available [16]. However, we focus on mixture regression modeling on a count data problem using Poisson distributions. Application was done on Cleveland Clinic Foundation heart disease data under different Poisson mixture models and results analysed with a version of the Zero Inflated Poisson Mixture Regression model also included and analysed.

## 2. Methods

### 2.1. Mixture Regression Model

In regression unobserved heterogeneity occurs if important covariates have been omitted either in the data collection or in some way such that important features are not accounted for in analysis, leading to biased parameter estimates. Mixture regression models seek to accommodate these different heterogeneity problems by grouping homogeneous observations into groups via a model based clustering. They can be standard, saturated, or with concomitant variables. The difference among these mixture regression models depends mainly on how the posterior probabilities are computed for the indicator variable. It has been shown that whilst standard variable models group and are classified based on the discriminant analysis rule and specify the joint distribution of the mixture distribution, the concomitant one is classified based on the logistic regression type rule and specifies the conditional distribution of response given the covariate [17]. In reality we tend to choose models with the best parameters compared to others.

Relaxing the normality and homogeneity assumptions in parameter distribution is quite possible by using mixture regression models especially using a GLM formulation as studied by Grun and Leish [18]. In a finite mixture regression model we consider a mixture of *k* components where each of the components follows a certain parametric distribution. Every component has a weight and prior probability for an observation to come from the given component and the mixture distribution is given by the weighted sum over the *k* components. Weights may depend on other variables not provided by data which are referred to as concomitant named here as concomitant variable mixture model. Traditionally, age and sex have been used to relate different segments of a population although they do not perform well in a prior segmented environment; thus the use of mixture models can be incorporated to improve classification in such segmented cases [19, 20]. Finite mixture of GLM format relaxes the assumption that regression coefficients and dispersion parameters are the same for all observations, giving a high degree of heterogeneity which can be incorporated into the clustering model.

### 2.2. Mixture Regression Classes

Two classes of mixture regression models were here considered: namely, the standard and concomitant variable mixture regression models. A component is here described by a GLM with different linear operator but the same error and link function resulting in a general standard mixture regression model of a GLM is given by

(1) $$\begin{array}{c} f\left(\;y\;|\;x,\Phi \;\right)=\underset{k}{\sum }\pi_{k}f_{k}\left(\;y\;|\;x;\beta_{0k},\beta_{k}\;\right), \end{array}$$

where *y* is the response variable assumed to follow a distribution of the exponential family, conditional upon component *k*, whilst the conditional expectation of the response variable is given as

(2) $$\begin{array}{c} E\left(\;y\;|\;x\;\right)=g^{-1}\left(\;\beta_{0k}+x^{\prime }\beta_{k}\;\right) \end{array}$$

with *g*(·) some link function. Here Φ = {*β*_0_, *β*, *π*}, *β*_0_ = (*β*_0*k*_), and *β* = (*β*_*k*_). Exponential family probability distributions are the commonly used and these include Poisson, binomial, normal, negative binomial, and geometric. We are going to concentrate on the Poisson due to the nature of the counts within the data to be considered. Often it is assumed that the component specific densities are from the same parametric family for each component; that is, *f*_*k*_ = *f* for notational simplicity and that the link function is also the same for all components *g*_*k*_ = *g*. In a cluster-wise regression setting this will be an obvious model choice as no prior knowledge about differences in distributional families of the components is available.

Another popular extension is to have a so-called concomitant variable model for the prior class probabilities, such that the weight *π*_*k*_ also depends on a set of explanatory variables (e.g., using a multinomial logit model). In such models substantial changes in the parameter estimates with quite small changes in the mixing model are a possible way to conclude that the random effects do not have enough information in the data about the distribution (i.e., there are heterogeneity issues). A concomitant variable mixture regression model is based on regression model (1) but uses a parameterisation on the posterior probabilities which assume that the concomitant variables fall into two sets *x* = (*x*_1_, *x*_2_) but the two sets may (partially or completely) overlap. The first set affects *y* whilst the second affects latent group indicator *z* variable assumed multinomial (i.e., *f*(*z*∣*x*; *π*) = ∏_*k*_ *π*_*k*∣*x*_). Thus the marginal distribution of the response variable is given as

(3) $$\begin{array}{c} f\left(\;y\;|\;x_{1},x_{2};\Phi \;\right)=\underset{k}{\sum }\pi_{k\;|\;x_{2}}f_{k}\left(\;y\;|\;x_{1};\beta_{0k},\beta_{k}\;\right). \end{array}$$

Here Φ = {*β*_0_, *β*, *γ*_0_, *γ*}, *β*_0_ = (*β*_0*k*_), *β* = (*β*_*k*_), *γ*_0_ = (*γ*_0*k*_), and *γ* = (*γ*_*k*_). The *γ*_*k*_'s are derived from the component indicator function

(4) $$\begin{array}{c} \pi_{k\;|\;x}=\frac{\exp\left(\gamma_{0k}+x^{\prime }\gamma_{k}\right)}{\sum_{h}\exp\left(\gamma_{0h}+x^{\prime }\gamma_{h}\right)} \end{array}$$

and are normally taken to be zero for identification. The component intercept parameter *β*_0_ can sometimes be aliased with the model intercept resulting in big standard errors for the intercept.

### 2.3. The Poisson Mixture Regression Model

A Poisson regression model is an example of a GLM in which distribution of the response *Y* with covariate vector *x* has Poisson density function given as

(5) $$\begin{array}{c} f\left(\;y,\lambda \;\right)=\frac{e^{-\lambda }\lambda^{y}}{y!}I_{A}\left(\;y\;\right). \end{array}$$

In (5), *E*(*Y*) = *λ*, the link function *g*(*λ*) = log⁡(*λ*) = *β* ^*T*^ *x*, *A* = {0,1, 2,…} is the set of nonnegative integers, and *I*_*A*_(*y*) is the indicator function, which is one if *y* belongs to the set *A* and is zero otherwise. The variance var⁡(*Y*) = *E*(*Y*) = *λ* and there exist dispersion problems when either var⁡(*Y*) > *E*(*Y*) (overdispersion) or var⁡(*Y*) < *E*(*Y*) (underdispersion). In many problems overdispersion is more common than underdispersion. Analysis of count data that are overdispersed relative to the Poisson distribution has been studied extensively; it is also known that application of the standard Poisson model may result in misleading inferences. There is therefore need for alternative methods of analysis to deal with the extradispersion problem. Finite mixture models are appropriate when the extra variability comes from the unobserved heterogeneity of the population, which composes two or more subgroups mixed in various proportions [13, 18, 20, 21]. In particular, Poisson mixture regression can be fitted to such extradispersed count data in the presence of covariate information. A mixture Poisson regression model assumes the objectives are drawn from a finite mixture Poisson where these distributions differ in intercept and coefficient of the explanatory variable in the regression component or are suspected to be heterogeneous. Mixture GLM models assume the count outcomes are clustered or represent repeated measurements and random effects that can be incorporated within the regression model to account for the inherent correlation between observations. Poisson mixture regression models which include the Poisson mixture model, negative binomial (NB), hurdle, Zero Inflated Poisson (ZIP), and Zero Inflated Negative Binomial (ZINB) models cater for different dispersion parameters and heterogeneity aspects, [22]. In particular the ZIP and hurdle models account for both heterogeneity and the extra zeros in the data.

A finite mixture model based on Poisson distribution may be written as model (1) where

(6) $$\begin{array}{c} f_{j}\left(\;y_{i},\theta_{ij}\;\right)=\frac{e^{-\lambda_{ij}}{\left(\lambda_{ij}\right)}^{y_{i}}}{y_{i}!}I_{A}\left(\;y_{i}\;\right). \end{array}$$

In (6) log⁡*λ*_*ij*_ = *β*_*j*_^*T*^ *x*_*i*_, *i* = 1 : *n*, *j* = 1 : *k*, and for the *k* component mixture

(7) EYi∑i=1kπjλij,var⁡YiEvar⁡Yi ∣ Zi+var⁡Yi ∣ Zi=EYi+vij,

where *λ*_*ij*_ = exp⁡(*β*_*j*_^*T*^ *x*_*i*_) is the mean of the *i*th response condition to its membership of the *j*th component of the mixture, *v*_*ij*_ = ∑*π*_*j*_ *λ*_*ij*_^2^ − (∑*π*_*j*_ *λ*_*ij*_)^2^, and *Z*_*j*_ is the component indicator vector of zeros and ones with *Z*_*ij*_ = (*Z*_*j*_)*i* which is one if it is from component *i* and zero otherwise. *v*_*ij*_ = 0, iff *λ*_1*i*_ = *λ*_2*i*_ = ⋯*λ*_*ki*_, that is, if the means of all components are the same, an indication that mixture model copes better than the homogeneous model. Heterogeneity across individuals in a Poisson mixture regression model is tackled in two ways which includes formulation in such a way that mean event rate has a discrete mixture distribution (i.e., varying across a finite number of unobserved classes) and that the mean event rate within each class depends on the explanatory variables. The general Poisson mixture regression model then assumes the following:

(i) Unobserved mixing process can occupy any of the *k* components, where *k* is finite and unknown.
(ii) For each of the observed count *y*_*i*_, there exists an unobserved random variable *Z*_*j*_ representing component at which *y*_*i*_ is generated and (*Y*_*i*_, *Z*_*i*_) are piecewise independent.
(iii) *Z*_*j*_ follows a discrete distribution with *k* points of support *P*(*Z*_*j*_ = *j*) = *π*_*j*_, with ∑*π*_*j*_ = 1.
(iv) Conditional on the *Z*_*i*_, *Y*_*i*_ follows a Poisson distribution.

Here *y*_*i*_, *i* = 1 : *n*, represent the *i*th response variable such that *y* is Poisson (*λ*_*ij*_) with probability *π*_*j*_(*x*), which are the mixing weights, where *x* and *z* are matrices and *k* is the number of components in the model. When discriminant operator using the normal log link function is used to separate observation we got standard Poisson mixture regression model.

Using logit and log-linear links to model *π*_*k*_(*x*) and *λ*_*ij*_, which denote vector of the regression coefficients of the *j*th component, respectively, gives

(8) logit⁡πjxlog⁡πjx1−πjx=xi′βj=1,x2k,xnkβ0j,β1j′′;

(9) log⁡λijzjTγj

for *β*_*j*_ and *γ*_*j*_ unknown parameters. This is a presentation of the concomitant variable Poisson mixture model. Whilst the covariates of the Poisson rates *z* are restricted to be the same, covariates in the mixing proportions (*x*) are not restricted to being the same as those in the Poisson rates (*z*). In Poisson model, dispersion problems can be modified via a different link function, alternative frequency distribution, or both. However, there are times when overdispersion or heterogeneity problems do not seem to be eliminated and hence can be rescued by mixture models. These assume a mixture of distribution as stated before such that

(i) if *k* = 1 we obtain the Poisson regression model reducing to Poisson model if *β*_11_ is specified to null vector,
(ii) if *β*_1*i*_ is specified to zero vector we obtain a *k* component Poisson mixture model,
(iii) if *k* = 2 and *λ*_1*k*_ = 0, for all *j*, then we obtain a Poisson regression model with extra weight at *y*_*i*_ = 0.

Thus mean and variance can be determined depending on the applicable condition. Maximisation of this Poisson mixture model is done using expectation and maximisation algorithm famously known as the EM. Extensive work on the EM algorithm and how it works have been studied for long by many authors including McLachlan et al. [13, 21]. As such EM algorithm explanations will not be considered here.

### 2.4. Zero Inflated Poisson Mixture Model (ZIPMR)

ZIPMR serves when the data involved has so many zeros which originates from how the counts are being generated in the model. It serves as a special mixture regression model to cater for both heterogeneity and zeros. It has a mixture of binary distribution that is degenerate at zero and an ordinary count distribution which can be Poisson or negative binomial. It considers the zeros to be separate from the nonzero, although it can include some zeros in the analysis with nonzeros. Zero inflated models can be thought of as finite mixture models (i.e., where two data-generating mechanisms are supposed, one generating 0s and one generating the full range of counts) [15]. It has been applied in many problems including epidemiology, occupational health, and children growth development [12]. In our case the Zero Inflated Poisson (ZIP) model was considered since there were structured zeros within the data considered indicating that they are true zeros from the counting process and zeros which came from dichotomising processes done during sampling. The ZIP model assumes that

(10) $$\begin{array}{c} y_{i}=\left\{\begin{array}{cc} 0, & if\;\;s_{i}=0\\ Poisson\left(\lambda_{i},\beta ,\gamma \right), & if\;\;s_{i}=1, \end{array}\right. \end{array}$$

where *s*_*i*_ represents whether component *i* has heart disease or not and other parameters are as defined before.

## 3. Application

### 3.1. Data

The study used Cleveland Clinic Foundation heart disease data set available at http://archive.ics.uci.edu/ml/datasets/Heart+Disease. It consists of 76 raw attributes and 303 observations for each attribute of which only 14 attributes were used for this research as given by Detrano [23]. The response variable (given as num) is a count of the rate at which an observation is diagnosed with heart disease where zero indicates no disease whilst 1, 2, 3, and 4 are the different levels of the heart disease stage. Our focus, however, will be to predict heart diseases diagnosis rate via a model based clustering approach using Poisson mixture regression models. This is justified by the fact that our data choice has mixture of both categorical and continuous variables with the response variable being count which is heterogeneous piecewise with the response being count. In this context, the Poisson mixture model assumes an underlying partition of the population into *k* homogeneous components, where each component has a rate of heart failure indicated by number of diagnosis on heart disease (angiographic disease status) dependent on the 13 different covariates.

### 3.2. Why Model Based Mixture Models on Heart Disease?

Data exploration here not shown results indicated that the data has lots of unexplained abnormalities. Most continuous variables showed skewed distributions and varying levels of high peaks indicating multimodality of the variables. The response variable relationship with indicators was observed to be nonlinear in most cases. Since the data is Poisson count, a general linear Poisson model was also analysed and results showed that it was underdispersed with a couple of other unexplained heterogeneity properties. As underdispersion is hardly addressed by change of link function or transformations, we introduce Poisson mixture models as a holistic approach to cater for the unexplained heterogeneity and extradispersion problems. Single parametric distributions on their own can never suffice to describe the data in a robust manner no matter how we transform the data. Irregularities of models as well as unobserved heterogeneity within the same model can be dealt with simultaneously in a single mixture models. This is because mixture models use a combination of both parametric density functions to represent each cluster and the overall population as mixture model thus capturing heterogeneity using several homogeneous groupings. Since a mixture model can capture these by way of dividing the observations into grouped distributions, we seek to employ a finite mixture model to heart disease data with so many explanatory variables causing heart problems and try to capture the heterogeneity in individuals via discrete mixture components. The source heterogeneity is here not known, so we have an incomplete data problem with assumed unknown number of homogeneous groups which will be determined via a finite mixture regression model. Our data has a count response variable and a mixture of both continuous and categorical predictor variables; thus we choose Poisson mixture regression model as appropriate for heterogeneity investigations as well as identifying the number of possible groupings.

## 4. Results

### 4.1. Finite Poisson Mixture Regression (FMPR) Model without Covariates

A GLM Poisson mixture model without covariates is here introduced in order to predict the heart disease. This is a Poisson mixture model that models the response variable, given the component membership of the observations is mutually independent. Flex mix package in R was used to determine different number of components (i.e., *β*_1*i*_ = 0). The summary results of the Poisson mixture model without covariates are shown in Table 1.

Table 1 shows the AIC and BIC values rounded to 3 significant figures for the Poisson mixture model without covariates under varying components, *K*. Based on both the AIC and BIC criteria a two-component Poisson mixture model best describes the data. We can therefore describe the heart disease diagnosis by a two-component mixture Poisson model.

#### 4.1.1. Two-Component FMPR Model without Covariates Properties

**Figure 1:**
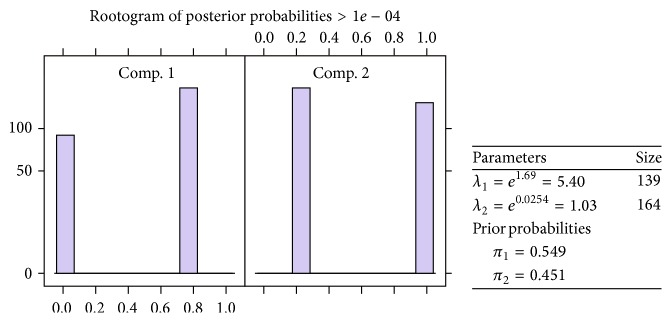
Information criteria graphs.

The properties of the chosen two-component Poisson mixture model without covariates are here summarised as Figure 1. The components from a Poisson mixture regression model without covariate are well separated as indicated by the rootograms in Figure 1 showing the posterior probabilities of the observation per given components. The prior probability of an observation getting to components one and two is 0.549 and 0.451, respectively. The mean component rate for diagnosis shows that component one has a high mean rate compared to component two.

### 4.2. FMPR with Covariates

A mixture Poisson regression model with the covariates is here analysed and applied to the heart disease data in order to predict the heart disease diagnosis using the 13 explanatory variables. Analysis was made using both the standard and a multinomial concomitant variable FMPR models and models compared with the GLM Poisson regression model (single component). The standard FMPR model has the single component Poisson mixture as the best model which correspond to the GLM Poisson model. It had a BIC value of 680 whilst all other models where above 700. The concomitant variable model supposes that if we let *y*_*i*_ denote the *i*th response, then the heart disease Poisson mixture regression model with 13 covariates assumes that each diagnosis count *y*_*i*_ is associated with at least one of the covariates given; diagnosis counts are independent and follow a mixture Poisson regression model with conditional means equal to the number of diagnoses. Finally, it also assumes that mean rate for the diagnosis is given by the exponential link function as a multiplicative factor *λ*_*ij*_ = *e* ^*β*_0*j*_+*β*_1*j*_*x*_1*j*_+⋯+*β*_*kj*_*x*_*kj*_^, where *x*_*ij*_ represent the *i*th observation in the *j*th component of predictor *x*.

**Figure 2:**
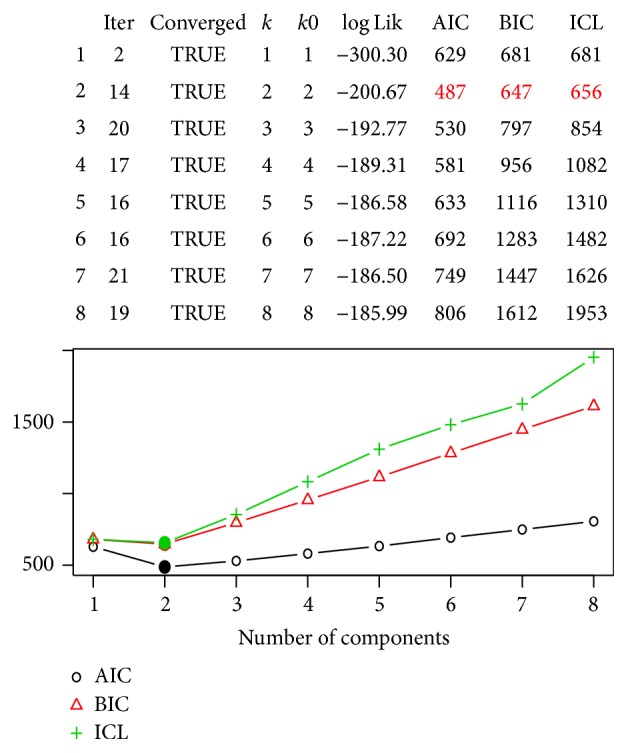
FMPR model selection: the Information criteria values and graph.

We also determined the number of components required for the concomitant FMPR model and results of the models considered and analysed are shown in Figure 2: both AIC and BIC model criteria seem to favour a 2-component FMPR model as shown by Figure 2 against even a single component (GLM Poisson). We therefore chose a two-component FMPR model as best and simple model due to its lowest BIC of 647 compared to all other mixture models including the standard FMPR and GLM Poisson models which has BIC value of 680.

#### 4.2.1. Two-Component Poisson Mixture Regression Results

**Figure 3:**
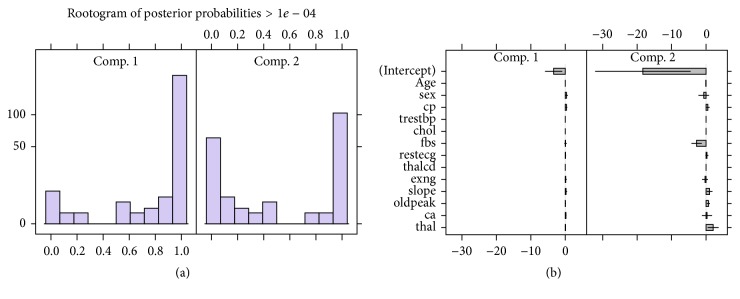
Poisson mixture regression plots: (a) concomitant FMPR model rootogram and (b) concomitant FMPR parameter CI.

Since the whole aim is to be able to predict heart disease, we analysed a Poisson mixture regression model with the already established 2 components for the data. A summary with parameter values and properties of the two-component Poisson mixture regression (FMPR) model is given as Figure 3.

Figure 3(a) shows the component distributions for the 2-component FMPR via the rootograms which shows that components are well separated. The parameter estimates confidence intervals for each component are shown as Figure 3(b). There seem to have wide confidence intervals for parameters in component 2 than they are in component 1. The model properties are summarised in Table 2 which shows that the 2-component FMPR model had 198 observations classified under cluster 1 at a probability of 0.642 whilst 105 are in cluster 2 with a prior probability of 0.358. These groups are mostly likely to be those who will develop the disease and those who will not.

#### 4.2.2. Parameter Estimates

The parameter estimates for the 2-concomitant variable FMPR model describe the average count rate for diagnosis for each component is given multiplicative exponential of the significant covariates within the component. Exponents of the parameters are shown in Table 3: the two-component FMPR models generally show that there is a general no change on the heart diagnosis rate with increases in age, cholesterol level (chol), maximum heart rate (thalacd), and resting blood pressure (trestbp) levels in component 1 whilst component 2 is not affected by age, trestbp, thalcd, and slope with a slight change due to cholesterol and restecg. The major risk factors of heart disease for component 2 include chest pain (cp), lack of exercises (exang), high fasting blood sugar (fbs), sex (males are at risk than woman), number of major vessels coloured by flourscopy (ca), oldpeak, and defect type (thal) whilst component 1 has main risk factors as resting electrographic results (restecg) and slope of peak exercise (slope). Analysis on the heterogeneity within a variable can also be inferred; for example, change in one unit increase in heart disease diagnosis has 56% increase more for males in component 2 than 1 compared to females. Therefore we are able to infer the differences not only between components but also within heart disease risks. Heart disease diagnosis can there be described differently for those in different components varying coefficients as well as risks that determine the rate of diagnosis.

### 4.3. Zero Inflated Poisson Mixture Regression (ZIPMR) Model Results

**Figure 4:**
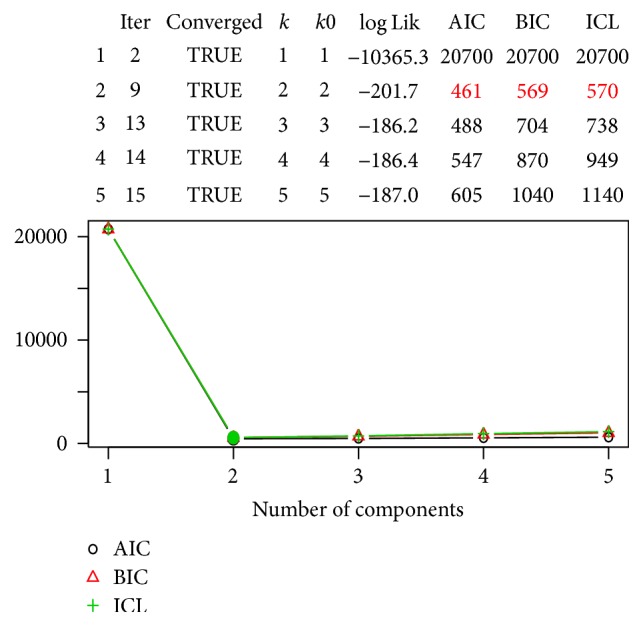
ZIPMR model selection.

The existence of so many zeros in the counts from the dichotomising process necessitated the use of ZIPMR model in the analysis. Results from the ZIPMR model under different components are given as Figure 4.

The best model criteria favour a two-component ZIPMR as best model as indicated by low AIC, BIC, and ICL values in Figure 4 compared to any other model including the GLM ZIP Poisson model which has the highest BIC value. ZIPMR model has one component which has no disease and the second component which responds to the heart disease diagnosis based on the multinomial covariates; thus a 2-component model best describes the heart disease prediction as those who are likely to get disease and those who will rarely develop the disease. The cluster and model properties of ZIPMR model are given in Table 4.

**Figure 5:**
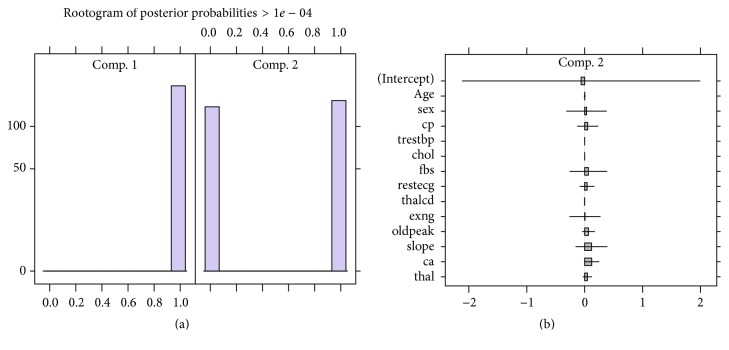
ZIP model results graphs.

Table 4 shows the AIC and BIC values for the ZIPMR model as well as component distribution where 164 observations are less likely to be diagnosed with heart disease at 54% rate in component 1 whilst there are 46% chances for 139 observations to get heart disease as described by component 2. The ZIPMR model seems to describe the heterogeneity and fit the data simpler and better than the normal 2-Poisson-mixture-component model due to its low BIC. The model also has highly separated components as figured by Figure 5(a) an indication of better separation among clusters. As component 2 describes those most likely to develop the disease, most parameters in the same component are also highly significant as indicated by the confidence intervals in Figure 5(b). Rating from the rootogram, the concomitant ZIPMR model has highly separated components as indicated by Figure 5(a).

#### 4.3.1. Parameter Values of the ZIPMR Models

To determine which predictor variables predict heart disease the most, we find the exponential model parameters as shown by the parameter values given in Table 5. The ZIPMR shows that the first component does not change with any variations of predictors as expected. It represents those without the heart disease, or who are not at risk with any of the variables given. However, most second component parameter values change with a change in the heart rate diagnosis. These outstanding risk factors include chest pains, lack of exercise, high fasting blood sugar levels, exercise slope, number of vessels, and defects state. Thus we are able to predict the heart disease diagnosis rate using a 2-component Poisson mixture model with a degenerate at zero. We can safely conclude that a change in fasting blood sugars levels or chest pain or exercise slope can contribute to rate at which one develops heart disease. Nine of the 13 explanatory variables which include sex, cp, fbs, restecg, exercise, slope, oldpeak, ca, and thal seem to give more risk in heart prediction than cholesterol, age, and maximum heart rate.

## 5. Discussion

### 5.1. Use of Finite Mixture Regression Models

The use of GLM mixture Poisson regression models was reviewed under different classes which include standard and concomitant variable models. The general use of the mixture regression models was to capture the heterogeneity which cannot be catered for via normal general Poisson regression models, by clustering homogeneous observations in components. This seemed to add a lot of value on the concomitant model than it does on the standard mixture model. Inclusion of mixture regression models greatly improves the model fit on the data as indicated by very low BIC values compared to GLM models. Thus improving on the efficiency at which we can predict heart disease since different components has different risk factors or predictors individuals can be diagnosed into components depending on whether they are major risk or minor risk factors. Mixture models also capture the extra variability via a concomitant variable Poisson mixture regression model, which tend to capture more heterogeneity and hence a better model than both the standard Poisson mixture regression model and the single component (general Poisson regression model here not shown). The presence of so many zeros within the count data also triggered us to incorporate a Zero Inflated mixture Poisson regression model to handle the many zeros for Poisson models. The idea of handling the zeros in a special manner as expected improves the models as it not only does fit the data properly but also made a reality assumption of putting those who are classified not to have the disease into a single cluster whilst those with disease in another component. The assumption is that those with the disease are modeled by Poisson mixture regression to predict the diagnosis allowing variability on the covariates. We noted that the Zero Inflated Poisson mixture model with a concomitant variable is even better than just a two-Poisson-mixture regression model as it has better and lower BIC and AIC values. Thus mixture models give more insight and better describe the data than supervised classification method does above its ability to efficiently predict heart disease and identify major risk factors.

### 5.2. Risk Factors

Although most heart disease prediction models assume that cholesterol, lack of exercise, and blood pressure are the main risk factors, we deduced that Poisson mixture regression models explain also the different levels at which these risk factors affect heart disease together with other correlated risk factors within the 2 heterogeneous groups involved in heart disease. On the contrary when Poisson mixture regression models are used, it showed that males are at more risk than females for heart disease whilst cholesterol does not seem to be a dangerous risk factor on heart diseases. Chest pain is also indicated among the major risk factors with nonanginal pain associated with heart disease more than angina pains, lack of exercise as before attributed to heart disease, those with abnormal resting electrocardiographic level who are at higher risk, the type of defects on the chambers, and number of major vessels an individual has. Therefore mixture models do not only fit the data better but also give more information on the heterogeneous characteristics of different individuals in the prediction of heart diseases. We can safely conclude that heart disease can be efficiently predicted by a two-component Poisson mixture regression model, with each component describing attributes differently. Thus the different components cater and captures the different heterogeneity properties exhibited by the general Poisson regression model hence the conclusion that model based clustering adds value to the prediction of heart diseases. The mean heart diagnosis is here given as a multiplicative factor of the parameter estimates and is differently predicted by two-component concomitant Poisson mixture regression model, with the best model catering for the zeros in ZIP mixture regression model.

## 6. Conclusion

Mixture model helps inference on the different groupings not only within the sample but also within each of the groupings. Heart disease can therefore be effectively diagnosed in an individual depending on the sex, whether they do exercise or not, and level of chest pain with a two-component Poisson finite mixture regression model. Hence heterogeneity across components is explained via different regression parameters and mixing probabilities. The risk factors can also be efficiently identified within each group (component) and individuals depending on the component.

## Figures and Tables

**Table 1:** Summary for FMPR models without covariates.

*K*	1	2	3	4	5
AIC	865	**797 **	801	805	809
BIC	868	**808**	819	831	842

**Table 2:** Summary of the FMPR model.

Model	DF	AIC	BIC	Prio.Prob.	Size
FMPR	43	490	650	*π*_1_ = 0.642	198
				*π*_2_ = 0.358	105

**Table 3:** Parameter estimates values.

FMPR model with concomitant		
	Comp. 1	Comp. 2
coef.(intercept)	0.867	7.33*e* − 07
coef.Age	1.00	9.93*e* − 01
coef.sex	1.07	1.64
coef.cp	1.10	8.45
coef.trestbp	0.998	1.00
coef.chol	0.10	1.01
coef.fbs	1.03	1.50
coef.restecg	1.09	1.05
coef.thalcd	0.998	9.97*e* − 01
coef.exng	1.19	3.33
coef.oldpeak	1.07	1.39
coef.slope	1.06	9.18*e* − 01
coef.ca	1.13	1.40
coef.thal	1.07	1.25

**Table 4:** Summary of the ZIPMR models.

Model	AIC	BIC	Prior prob.	size
ZIPR_1_	462	569	*π*_1_ = 0.54	164
			*π*_2_ = 0.46	139

**Table 5:** ZIPR models estimated parameter values.

ZIPR with concomitant		
	Comp. 1	Comp. 2
coef.(intercept)	0	0.120
coef.Age	1	1.00
coef.sex	1	1.28
coef.cp	1	1.24
coef.trestbp	1	1.00
coef.chol	1	1.00
coef.fbs	1	1.04
coef.restecg	1	1.08
coef.thalcd	1	0.997
coef.exng	1	1.12
coef.slope	1	1.23
coef.oldpeak	1	1.10
coef.ca	1	1.23
coef.thal	1	1.12
